# High White Light Photosensitivity of SnSe Nanoplate-Graphene Nanocomposites

**DOI:** 10.1186/s11671-017-2021-0

**Published:** 2017-04-07

**Authors:** Jinyang Liu, Qingqing Huang, Kun Zhang, Yangyang Xu, Mingzhu Guo, Yongqiang Qian, Zhigao Huang, Fachun Lai, Limei Lin

**Affiliations:** 1 College of Physics and Energy, Fujian Normal University, Fuzhou, 350117 People’s Republic of China; 2 Fujian Provincial Key Laboratory of Quantum Manipulation and New Energy Materials, Fuzhou, 350117 People’s Republic of China; 3 Fujian Provincial Collaborative Innovation Center for Optoelectronic Semiconductors and Efficient Devices, Xiamen, 361005 People’s Republic of China; 4 Center for Micro- and Nanoscale Research and Fabrication, University of Science and Technology of China, Hefei, Anhui 230026 People’s Republic of China

**Keywords:** Photodetector, SnSe, Nanoplate-graphene, Nanocomposites, Photosensitivity, 68.65.-k, 81.15.Gh, 78.67.-n

## Abstract

The multi-functional nanomaterial constructed with more than one type of materials has gained a great attention due to its promising application. Here, a high white light photodetector prototype established with two-dimensional material (2D) and 2D nanocomposites has been fabricated. The 2D-2D nanocomposites were synthesized with SnSe nanoplate and graphene. The device shows a linear *I-V* characterization behavior in the dark and the resistance dramatically decreases under the white light. Furthermore, the photosensitivity of the device is as large as 1110% with a rapid response time, which is much higher than pristine SnSe nanostructure reported. The results shown here may provide a valuable guidance to design and fabricate the photodetector based on the 2D-2D nanocomposites even beyond the SnSe nanoplate-graphene nanocomposites.

## Background

Atomically thin two-dimensional (2D) materials, including graphene [1–3], BN [4], Be_2_Se_3_ [5], MoS_2_ [6], GaSe [7], SnSe [8], and SnSe_2_ [9], have attracted great attention due to their unique properties distinguished from the bulks in the past decades. However, the single function of the material has become the bottleneck for their further application. As a result, building blocks in nanocomposites constructed with two or even more components have provided a new strategy. In particular, the nanocomposites constructed with the graphene have become a great interest. These nanocomposites not only preserve the favorable properties of graphene and the other components but also greatly enhance the intrinsic properties due to the synergetic effect between them. At present, three types of the composites based on graphene have been constructed: (i) 0D-2D nanocomposites, for example, ZnO NP-RGO [10], CdSe NP-RGO [11], PbS QD-graphene [12], and GeSi QD-graphene [12]; (ii) 1D-2D nanocomposites, for example, PbSe nanorod-graphene [13] and ZnO nanorod-graphene [14]; (iii) 2D-2D nanocomposites, for example, GaSe nanosheet-graphene [15], few-layer InSe-graphene [16], and CdSe nanosheet-graphene [17]. Among them, due to the larger interface region from the face-to-face contact comparing to the point-to-face contact in the 0D-2D nanocomposites and the line-to-face contact in the 1D-2D nanocomposites, the 2D-2D nanocomposites are suggested to be the most promising materials in photodetector, photocatalysis, energy storage and conversion, sensor, and so on.

In this report, the 2D-2D nanocomposites namely SnSe nanoplate-graphene nanocomposites were synthesized. As an important p-type semiconductor, SnSe has attracted intense attention in solar cells, photodetectors, and near-infrared optoelectronic devices due to its narrow band gap (~0.90 eV indirect and ~1.30 eV direct), earth-abundance, less toxicity, and chemical stability [18–20]. Then, a white light detector prototype based on the as-synthesized products was constructed after a series of characterization by XRD, SEM, and Raman. The device shows a linear *I-V* characterization behavior in the dark, and the resistance dramatically decreases under the white light. The photosensitivity of the device is as large as 1110% with a rapid response time. The results shown here may provide a valuable instruction to design and fabricate photodetectors based on graphene and even extend to other 2D-2D nanocomposites.

## Methods

### Chemicals

All chemical materials were of analytical grade and used as received without further purification. Tin (II) chloride dihydrate (SnCl_2_·2H_2_O; ≥98%), polyvinylpyrrolidone (PVP; 99.0%), and benzyl alcohol (≥98%) were purchased from Tianjing Fuchen Chemical Reagents Factory. Selenium dioxide (SeO_2_; 99.9%) were obtained from Chengdu Ai Keda Chemical Technology Co., Ltd. Graphite oxide (GO) was prepared through Hummers method [21]. Some common organic solvents (ethanol and so on) were of analytical grade and obtained from Sinopharm Chemical Reagent Co., Ltd.

### Synthesis of the SnSe Nanoplate-Graphene Nanocomposites

In a typical synthesis of the SnSe nanoplate-graphene nanocomposite, SeO_2_ (0.8 mmol/L), SnCl_2_·2H_2_O (0.8 mmol/L) and poly (vinyl pyrrolidone) (PVP; 0.32 g/mL), GO (0.075 g/mL) is added into benzyl alcohol (20 mL) at room temperature. The mixed solution was transferred into a three-neck round-bottom flask, sealed, and then degassed with pure N_2_ (99.99%) under magnetic stirring. Then the mixture was heated up to 200 °C and allowed to be aged for another 12 h at this temperature in N_2_ atmosphere. Finally, the solution was cooled down to room temperature naturally, and the products were obtained by centrifugation at 10,000 rpm for 10 min to purify at least twice by re-suspending them into absolute alcohol. The products could then be well re-suspended in ethanol for further characterization.

### Characterization

The powder X-ray diffraction pattern (XRD) is characterized by PANalytical X’Pert ProMPD (Cu Kα, *λ* = 0.15418 nm). The UV-Vis-NIR was characterized by the Perkin Elmer Lambda 950. The scanning electron microscopy (SEM) was characterized by Hitachi SU-8010. Transmission electron microscopy (TEM) attached with selected area electron diffraction (SAED) was characterized by JEOL ARM-200F. The Raman spectroscopy was recorded at room temperature using HORIBA Jobin Yvon Evolution with laser excitation at 532 nm with power less than 5 mW. The interdigital gold electrode (the separation between them was 5 μm) on the SiO2 substrate was fabricated by photolithography with lift-off technology. The electronic property was measured by Keithley 4200 semiconductor characterization systems. The SAN-EI ELECTRIC XES-40S1 Solar Simulator (100 mW/cm^2^) was used as the white light source.

## Results and Discussion

### Crystal Structure

**Fig. 1:**
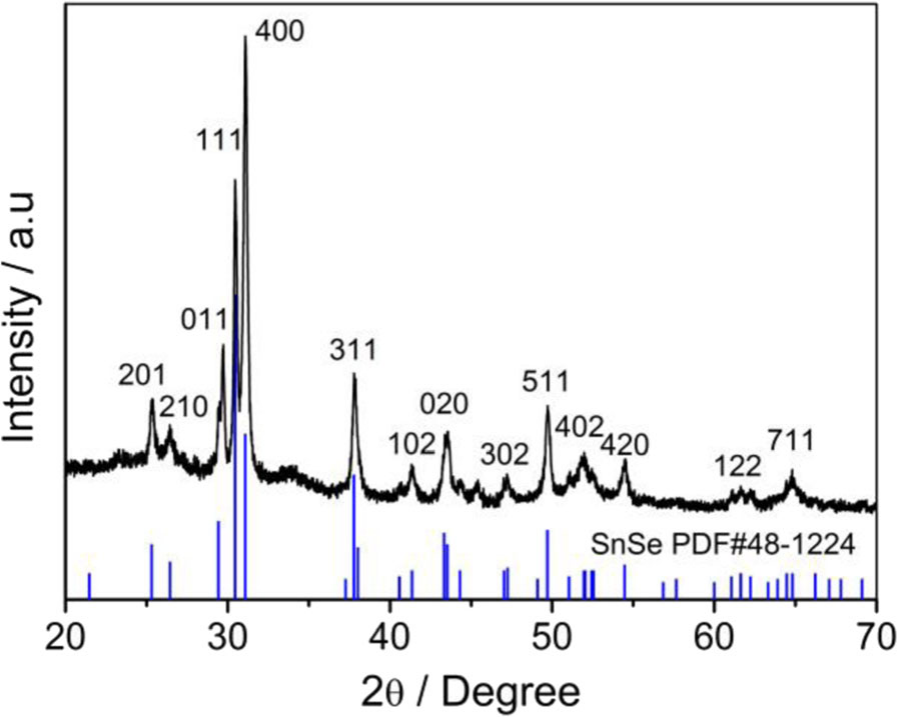
The XRD pattern of the SnSe-graphene nanostructure

The powder X-ray diffraction (XRD) pattern is performed to elucidate the phase structure of the SnSe nanoplate-graphene nanocomposites. As shown in Fig. 1, all of the diffraction peaks could be indexed to the orthorhombic SnSe structure with cell unit of *a* = 11.50 Å, *b* = 4.15 Å, and *c* = 4.44 Å (PDF #48-1244 Pnma(62)), which is consistent with the previous reports [8, 22]. It is worth-noting that the predominant peaks are (400), which indicate highly orientation of the SnSe nanoplates grown along (100) zone axis.

### Morphology and Raman Spectroscopy

**Fig. 2:**
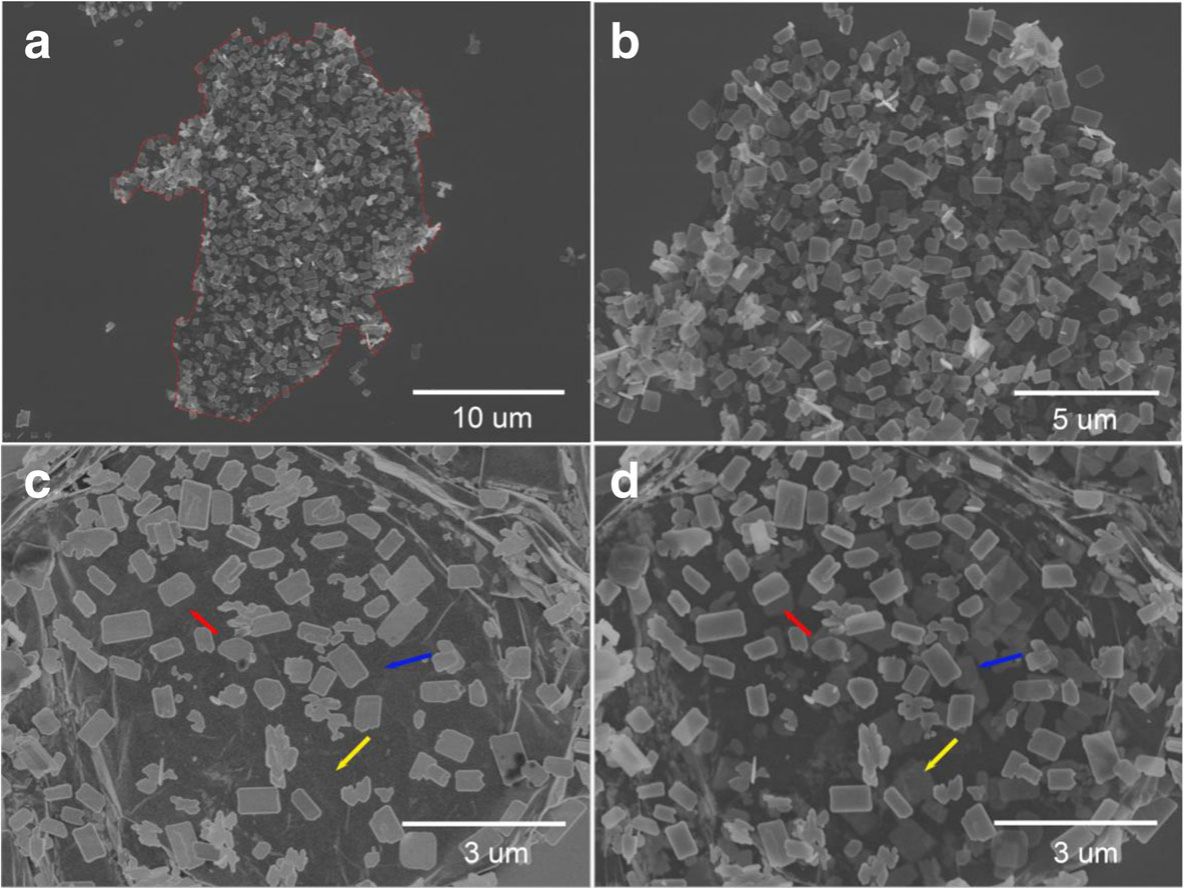
**a**, **b** The low-magnification and high-magnification SEM image of SnSe nanoplate-graphene nanocomposites; **c**, **d** the SEM image of SnSe nanoplate-graphene nanocomposites characterized with the accelerated voltage of 1 and 5 kV, respectively

The as-synthesized products were then characterized by SEM and the typical results are shown in Fig. 2. The majority of the SnSe nanoplates were rectangular with a typical lateral size of 1–2 μm, anchoring to the graphene sheet. The SnSe nanoplate-graphene nanocomposites are about ~10 μm, which are consistent with the GO precursor. The graphene sheet is ambiguous in the low-magnification SEM image due to the single atomic layer thickness; however, its wrinkle is observed in the high-magnification SEM image as shown in Fig. 2c, d. Through detailed examination of the samples, it can be found that the SnSe nanoplates have well size distribution and excellent dispersion on the graphene sheet. An interesting phenomenon was observed in the SEM characterization, only the SnSe nanoplates on the upper surface are able to be observed in the low accelerated voltage (1 kV) as shown in the Fig. 2c; however, in the high accelerated voltage (5 kV), the SnSe nanoplates on the bottom face can also be viewed clearly as shown in Fig. 2d. Consequently, it offers a novel method to distinguish the position of the SnSe nanoplates on graphene sheet, and it may be extended to characterize the similar structure. Therefore, it can be concluded that the SnSe nanoplates decorate on both sides of the graphene sheet.

**Fig. 3:**
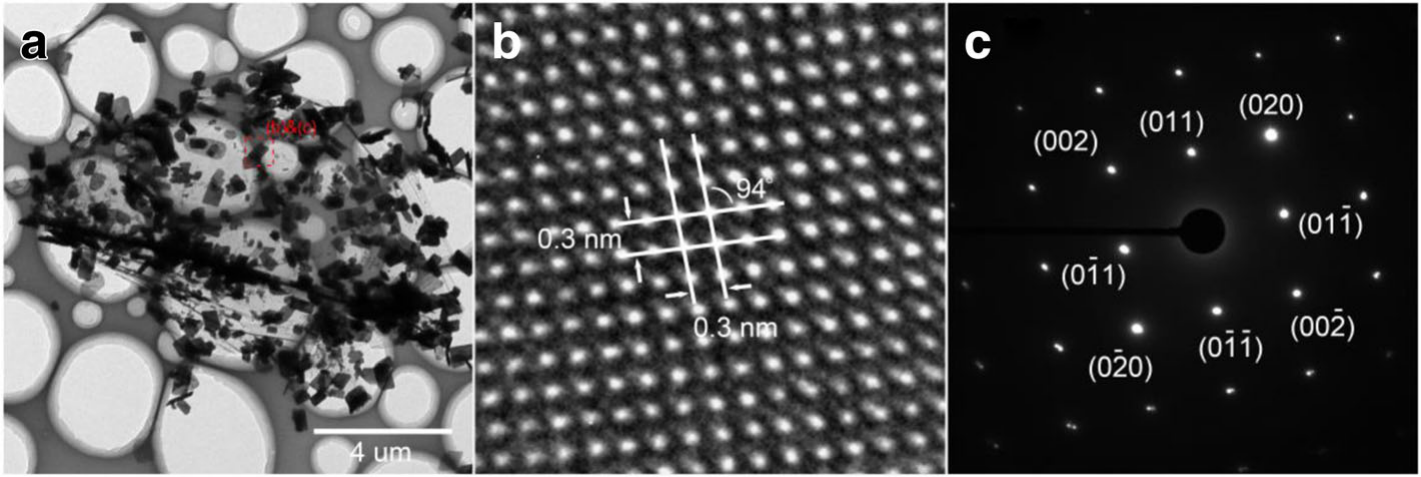
**a** The low-magnification TEM image of the representative SnSe nanoplate-graphene nanocomposites; **b**, **c** the high-magnification TEM image and SAED pattern of the representative SnSe nanoplate of the rectangular of **a**, respectively

In order to obtain the detailed crystal structure and crystal quality of the as-synthesized products, the SnSe nanoplate-graphene nanocomposites were characterized by transmission electron microscopy (TEM), HRTEM, and the selected-area electron diffraction (SAED). As shown in Fig. 3a, the graphene lay flat on the TEM grids, and the SnSe nanoplates dispersed uniformly on the graphene sheet, which are consistent with the SEM results illustrated above. The SnSe nanoplates and graphene are relatively stable against the high energy electron beam irradiation in a TEM. In addition, the HRTEM image of the square area masked in Fig. 3a shows clear orthogonal lattice fringes with both lattice spacing of ~0.30 nm. The intersection angle of the lattice fringes is approximated 94° (Fig. 3b), which is in good agreement with the angle between the planes of (011) and (0-11) of the orthorhombic SnSe crystal structure [22]. Furthermore, the SAED data (Fig. 3c), taken from the individual SnSe nanoplate, exhibits a clear orthogonally symmetric spot pattern, indicating the single-crystal nature of the sample. Therefore, we can conclude that the as-synthesized products show high quality, in which the SnSe nanoplate can be well anchored to and dispersed on the graphene sheet.

**Fig. 4:**
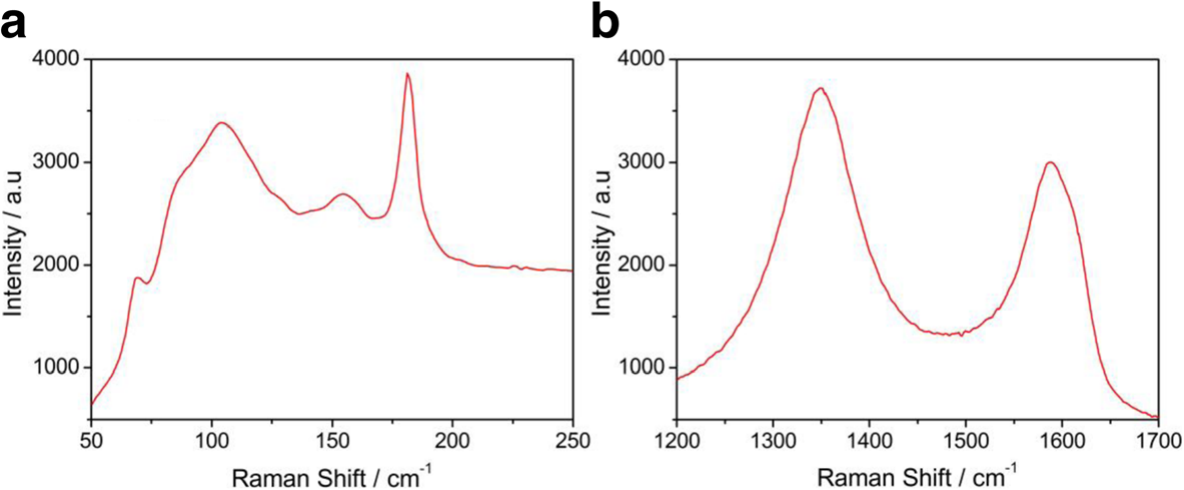
**a**, **b** The Raman spectrum of SnSe and graphene of the SnSe nanoplate-graphene nanocomposites

Furthermore, the Raman spectroscopy was also performed to investigate the quality of the SnSe nanoplate-graphene nanocomposites. As illustrated in Fig. 4a, four characteristic peaks at 69.1, 104.1, 154.4, and 181.0 cm^−1^ respectively are clearly observed, which are consistent with the previous report [8, 22]. The Raman peak at 104.1 cm^−1^ belongs to the B3g phonon mode originated from the rigid shear modes of a layer with respect to its neighbors in the c directions. It has the highest intensity, indicating the layer structure of the SnSe nanoplates, which is consistent with the XRD results shown above. On the other hand, the D band at approximately 1349.8 cm^−1^ originated from the disorder and the G band at 1588.4 cm^−1^ coming from the in-phase vibration were all observed clearly (Fig. 4b), which are consistent with the intrinsic property of the reduced graphite oxide (RGO). All these results shown above indicate that the SnSe nanoplate-graphene nanocomposites have a high quality.

### Absorbance Spectrum

**Fig. 5:**
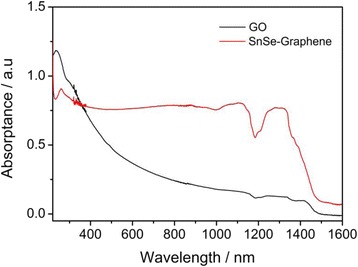
UV-vis absorption spectra of GO and the SnSe nanoplate-graphene nanocomposites

The optical absorption behavior of the SnSe nanoplate-graphene nanocomposites and GO dispersed in ethanol were studied by UV-Vis-NIR spectroscopy in the region of 400–1600 nm. As seen in Fig. 5, GO shows a strong absorption peak at 236 nm together with a weak shoulder peak at 301 nm, which is consistent with previous report [23, 24]. It is well known that the UV-vis spectrum of GO exhibits a dominant peak at 231 nm, corresponding to *π* → π* transitions of aromatic C–C bonds, and a weak shoulder near 300 nm, attributing to σ → *π* transitions of C–O bonds [25]. On the other hand, the SnSe nanoplate-graphene nanocomposites show a good absorption from the near infrared spectrum (~1470 nm), visible-light to the ultraviolet-light region; and a strong absorption peak originated from RGO was observed at 265 nm, which may be generally regarded as the excitation of plasmon of graphitic structure. The results indicate that the SnSe nanoplates and RGO really existed in the hybrid. Due to their wide range absorption region, the as-synthesized products have attracted intense attention for application in solar cells, photodetectors, and near-infrared optoelectronic devices.

### Photo-Electronic Property of the Device

**Fig. 6:**
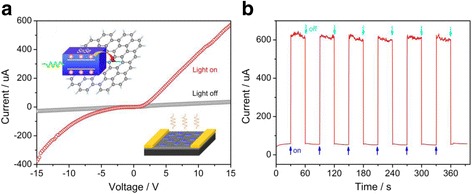
**a** The typical *I-V* characterization of the photodetector device constructed with the SnSe nanoplate-graphene nanocomposites, the schematic and structural diagram of the photodetector shown in the *left upside* and *right downside inset*; **b** the photosensitivity of the photodetector device with the voltage of 15 V under the *white light* or not

In order to evaluate photo-electronic property of the SnSe nanoplate-graphene nanocomposites for the white light, the photodetector device prototype was fabricated as described in the inset of Fig. 6a. The white light photodetector device was fabricated by drop casting the SnSe nanoplate-graphene nanocomposite alcoholic solution onto an interdigital gold electrode on the SiO_2_ substrate. The electrode was fabricated by photolithography, and the separation between the adjacent fingers was 5 μm. The white light response of the device was measured by Keithley 4200 apparatus under the periodical illumination of a sunlight simulator with a power density of 100 mW/cm^2^. The typical *I-V* characterization of the device is shown in Fig. 6a. The good linear behavior in the dark demonstrates the Ohmic contact between the Au electrode and hybrid material. However, it shows good linear behavior in positive voltage and non-linear behavior in the negative voltage under the white light. This phenomenon may come from the fact that one contact resistance is the ohmic contact and the other is the Schottky contact. The white light photoresponse current were also measured with a fixed bias of 15 V. The device shows an excellent performance as a white light photodetector, as demonstrated in Fig. 6b. First, the response to white light is reversible and stable, indicating the device is very robust. Second, the photosensitivity is much high. If we define the photosensitivity as the ratio of the current under the white light to that in the dark, it can be calculated to be as large as ~1110%. Third, the response is very quick. The response time of our device is only about 1 s and the recovery time can be less than 1 s. However, the performance of the devices fabricated with SnSe nanostructures were not very satisfactory in the past, for example, Zhao et al. [26] have grown the SnSe nanoplates by CVD and its photosensitivity is only about 140%; Li et al. [8] synthesized SnSe nanosheets through solution thermal reaction and constructed photodector device comprising the SnSe nanosheets and poly-(3-hexylthiophene) (P3HT) hybrid films and its highest photosensitivity is less than 200%. Therefore, the photosensitivity of our devices is at least five times larger than that previous reported [8, 26]. It may be understood that, upon exposure to white light, the electrons and hole carriers are first excited from the SnSe nanoplates and transferred to the RGO rapidly, considering the special energy band of SnSe nanoplates and RGO [11] and unique 2D-2D structure (as shown in the inset of Fig. 4a). On the other hand, the electrical conductivity of the SnSe is poor, while the electrical conductivity of the RGO is very good. Furthermore, as for the two atomically thin 2D materials, the SnSe nanoplates and RGO contain high specific surface area. And subsequently, the SnSe nanoplate-graphene nanocomposites possess larger interface region from the face-to-face contact between the SnSe nanoplates and graphene. Therefore, the RGO serving as the carrier collectors can transport the carriers to the electrodes more efficiently and rapidly. In this context, the interfacial design and control between the different components in the hybrid is very important, which is also the theme of the optoelectronic device and needed further understanding in the future.

## Conclusions

The 2D-2D nanocomposites namely SnSe nanoplate-graphene nanocomposites have been successfully synthesized and investigated. The photodetector device prototype of the as-synthesized products was fabricated and characterized carefully. The device shows a linear behavior in the dark and the resistance decreases sharply under the white light. Furthermore, the photosensitivity of the device is much larger than one order of magnitude with reversible and stable under a rapid response time, which is much better than previous reported. The high performance of the device may originate from the larger interface region from the face-to-face contact and special energy band of SnSe nanoplates and graphene. The results may hopefully be guidance to design and fabricate the photodetector based on the 2D-2D nanocomposites even beyond the SnSe nanoplate-graphene nanocomposites.
